# A secondary analysis of the effects of IQOS use on cigarette smoking: Do the effects differ by sex?

**DOI:** 10.1016/j.drugalcdep.2025.112848

**Published:** 2025-08-22

**Authors:** Janet Audrain-McGovern, Olivia Klapec, Ariadni Oikonomou, Priyanka Mistry, Joseph Smith, E. Paul Wileyto, Andrew A. Strasser

**Affiliations:** a Department of Psychiatry, Perelman School of Medicine, University of Pennsylvania, Philadelphia, PA, USA; b Department of Biostatistics and Epidemiology, Perelman School of Medicine, University of Pennsylvania, Philadelphia, PA, USA

**Keywords:** Cigarettes, Combustible, Heated tobacco products, IQOS, Sex differences

## Abstract

**Introduction:**

Through secondary data analyses, we investigated sex differences in the effects of IQOS, a heated tobacco product, on combustible cigarette smoking.

**Methods:**

Adults who smoke cigarettes (N = 118; 21–65 years old) completed a baseline ad-lib smoking period (days 1–5), two laboratory visits (days 6–7), and a 14-day period where they were instructed to switch from smoking cigarettes to using IQOS 3.0 (days 8–21). Models estimated the changes in cigarettes smoked per day (CPD) and the percentage of baseline CPD substituted by HeatSticks during the switch period. Bivariate statistics assessed sex differences in covariates and IQOS-associated effects (i.e., subjective reward, relative reinforcing value, craving relief, and withdrawal relief) for model inclusion.

**Results:**

Males and females significantly reduced their CPD to 29.8 % and 21.8 % of their baseline CPD by the end of the switch period, respectively. CPD slopes were significant for males (β =−0.46/day [CI95 % −0.97 to −0.04] p = 0.002) and females (β =−0.83/day [CI95 % −1.34 to −0.31] p < 0.001), although slopes did not significantly differ (p = 0.3). Males and females substituted HeatSticks at 83.2 % and 67.4 % of their baseline CPD across the switch period. The IQOS slopes were significant for males (β =1.136/day [CI95 % 0.59–2.14] p = 0.001) but not females (β =0.64/day [CI95 % −0.67–1.94] p = 0.34), although the slopes did not significantly differ (p = 0.36). There were no significant sex differences in IQOS-associated effects (all p values > 0.05).

**Conclusions:**

Males and females do not appear to differ in IQOS-associated effects or the initial substitutability of IQOS for combustible cigarettes.

## 1. Introduction

Combustible cigarette smoking is the leading preventable cause of premature morbidity and mortality, yet the last novel smoking cessation medication entered the market two decades ago. Systematic reviews have documented that smoking cessation outcomes for males who smoke are similar for nicotine replacement therapy (NRT), bupropion, and varenicline (Smith et al., 2017). However, females have lower cessation rates than males when using NRT or bupropion, and only varenicline supports comparable long-term cessation rates (Smith et al., 2017). Sex differences in response to smoking cessation medication are thought to be due in part to nonpharmacological effects (e.g., behavioral and sensory effects) motivating female’s cigarette smoking, while the pharmacological effects of nicotine drive male’s smoking (Jensen et al., 2016; Perkins et al., 2002, 2018). Studies have shown that subjective reward and relief from abstinence symptoms are tied more to the behavioral and sensory effects of smoking itself for females than males (Perkins, 2009; Perkins et al., 2002; Rose, 2006).

Males and females who have been unable or uninterested in quitting smoking using FDA-approved smoking cessation medications are increasingly turning to noncombustible alternative nicotine delivery products as cessation tools (Mayer et al., 2020; Zhu et al., 2017). Noncombustible tobacco products offer a potentially less harmful alternative means of nicotine delivery for those who would not otherwise quit smoking. Products that provide behavioral and sensory effects similar to smoking may offer another option for women who have been unable to quit smoking with varenicline or are disinterested in its use.

The heated tobacco product IQOS heats instead of burning pressed leaf tobacco sticks to produce a nicotine-containing aerosol for inhalation. Because IQOS heats rather than combusts tobacco, there is less toxicant exposure than combustible cigarette smoking (Simonavicius et al., 2019), supporting its reduced exposure product designation (U.S. Food and Drug Administration, 2020). Only two studies have examined the substitutability of IQOS for cigarette smoking. Thirty-nine percent (43/110) of a sample of adults from Catania, Italy, switched from combustible cigarettes to IQOS (CO < 10 ppm) after receiving 12 weeks of IQOS and smoking cessation counseling (Caponnetto et al., 2023). Similarly, our study of 90 U.S. adults showed that IQOS use resulted in a 70 % reduction in cigarette smoking, and confirmed that almost 20 % completely switched to IQOS after 14 days of IQOS provision without counseling (Audrain-McGovern et al., 2024). In addition, we documented that the positive reinforcing effects of IQOS fostered use and the transition away from combustible cigarette smoking while the relief of abstinence symptoms played little role.

Whether IQOS, a product that provides a similar behavioral and sensory experience to smoking, aids the transition away from cigarettes for females, as well as for males, has not been studied. Understanding sex differences in response to harm reduction products, such as IQOS, is critical given that females have fewer efficacious medications to quit smoking than males and, as a result, continue smoking cigarettes. The present study sought to determine whether there were sex differences in IQOS-associated effects (i.e., craving relief, withdrawal relief, subjective reward, relative reinforcing value), cigarette smoking, and the substitution of IQOS for combustible cigarettes.

## 2. Methods

### 2.1. Study participants

Participants were 118 adults 21–65 years old who smoked ≥ five cigarettes per day (CPD) for the past 12 months and had no plans to quit smoking in the next 30 days but were interested in quitting within six months. A carbon monoxide (CO) value of > 10 ppm was used to verify smoking status. Exclusion criteria included the regular use (≥ 5 days/past 30 days) of other nicotine products, current use of specific medications (e.g., smoking cessation medication, stimulants, opiates), consumption of > 25 standard beverages with alcohol beverages a week, regular recreational substance use (except cannabis), serious medical condition within the past year, self-reported psychiatric conditions involving psychosis, pregnancy, and lactation (Audrain-McGovern et al., 2024).

The study was conducted at the University of Pennsylvania and approved by the Institutional Review Board. Participants provided written informed consent before enrolling in the study. Recruitment began in September 2020 for a pilot project of 28 participants (DeAtley et al., 2024) and in September 2021, for the primary study involving 90 participants (Audrain-McGovern et al., 2024). The accrual was completed on July 24, 2023. This study is a secondary data analysis of the data collected in these two studies. The primary study is registered at CT.gov (NCT05076708).

### 2.2. Study procedures

Participants were recruited from Philadelphia, Pennsylvania, through social media advertising. Ad respondents who met inclusion and exclusion criteria via the telephone pre-screen provided in-person informed consent and completed an intake screening to document a negative urine drug screen, a negative urine pregnancy test (females only), a breath alcohol test of 0.000, and a CO value > 10 ppm. Participants eligible at the intake screening completed baseline measures and received instructions to smoke as usual for the next five days while collecting all spent filters each day (Days 1–5). The average daily spent filters served as the baseline smoking rate.

On day 6, participants arrived at the lab at 9 a.m. after 10 h of overnight cigarette smoking abstinence (CO verified <10 ppm). Participants completed self-report measures of craving and withdrawal. Then, participants completed two 14-puff IQOS HeatStick exposures, one menthol-flavored and the other tobacco-flavored, 45 min apart, with flavor order counterbalanced and stratified by sex (Audrain-McGovern et al., 2016, 2024). After each exposure, subjective reward, craving, and withdrawal were measured.

On day 7, participants arrived at the lab at 9 a.m. following overnight abstinence. Participants were introduced to a validated behavioral choice task, which assessed the reinforcing value of IQOS relative to cigarettes (Audrain-McGovern et al., 2016, 2024). Participants completed the task by moving a computer mouse to hit either an IQOS image or a cigarette image on one of two computer screens to earn points toward either IQOS puffs or cigarette puffs across 10 trials using a concurrent schedule. Consistent with relative reinforcement paradigms, earning puffs for IQOS required a fixed amount of work (FR-25, 25 target hits to earn each puff), while earning puffs for cigarettes required a progressively increasing amount of effort (PR-25x) to earn each puff. The task was performed until a participant completed 10 trials and accumulated a total of 10 points from which they could have earned either one puff of IQOS (up to 10 puffs) or one puff of a cigarette (up to 10 puffs) for each point collected, or a combination thereof.

Participants were then given the IQOS 3.0 system, a supply of Marlboro HeatSticks in their preferred flavor(s), and date-stamped zipped bags for the daily collection of used HeatSticks and spent cigarette filters. Participants were instructed to switch completely from smoking cigarettes to IQOS use for 14 days (days 8–21), beginning on the morning of day 8. Smoking cessation counseling was not provided as the sample enrolled was not seeking treatment or interested in quitting in the very near future. Participants were instructed to collect any spent cigarette filters if they did smoke beginning on the morning of day 8 until the end of day 21. Participants returned to the lab every three days to provide CO readings and return used HeatSticks and cigarette filters. Participants were compensated $500.

### 2.3. Measures

#### 2.3.1. Covariates and predictor variables

Biological sex and age were self-reported. Covariates included self-identified race and nicotine dependence as measured by the 6-item Fagerström Test of Nicotine Dependence (Heatherton et al., 1991).

##### Subjective Reward.

The subjective rewarding value of IQOS was measured by the 2-item satisfaction subscale from the Cigarette Evaluation Scale adapted for IQOS use, which asked “Was it satisfying”? and “Did it taste good”? (Adriaens et al., 2018; Stone et al., 2022).

##### Relative Reinforcing Value.

The reinforcing value of IQOS use relative to cigarette smoking was measured with a validated choice task, evaluating the preference for IQOS versus cigarette puffs (Audrain-McGovern et al., 2024). The relative reinforcing value of IQOS was determined by the breakpoint, which is the highest trial completed across 10 trials to earn cigarette puffs (Bickel et al., 2000). Relative to IQOS, 78 % of male and 83 % of female participants worked predominately for cigarettes (≥ 5 trials for cigarette puffs; (Epstein et al., 2004)), while 22 % of males and 17 % of females were far less willing to work for cigarette puffs (≤ 2 trials for cigarette puffs). As such, we also evaluated a dichotomous indicator.

##### Craving and Withdrawal Relief.

Craving was measured by summating the negative subscale (4 items, anticipation of negative reinforcement from smoking) and the positive subscale (5 items, anticipation of positive reinforcement from smoking) from the 10-item Brief Questionnaire of Smoking Urges (Cox et al., 2001). Withdrawal symptoms were measured by summating the 8-item Minnesota Nicotine Withdrawal Scale (Hughes et al., 1984; Toll et al., 2007). Relief scores were calculated as post-IQOS exposure responses minus abstinence-induced responses.

#### 2.3.2. Outcome variables

##### Cigarette Smoking.

The primary outcome was the count of cigarettes smoked per day (CPD) across the 14-day switch period (days 8–21) compared to the average CPD at baseline (days 1–5). This outcome was determined by counting each daily spent cigarette filter returned and self-reported CPD (r = 0.98) (Roulet et al., 2021). Daily counts were transformed to a percentage of the participant’s average baseline smoking rate.

##### IQOS Use.

Daily IQOS consumption during the 14-day switch period (days 8–21) was measured by counting each daily spent tobacco HeatStick (Roulet et al., 2021). The daily HeatStick counts were transformed to a percentage of the participant’s baseline smoking rate to quantify the percentage of cigarettes substituted by HeatSticks.

### 2.4. Statistical analyses

Longitudinal daily cigarette and IQOS HeatStick use were analyzed over the switch period, normalized to the mean smoking rate over the baseline period, and expressed as a percentage. GEE regression methods were used, assuming an exchangeable correlation and a Gaussian distribution family. However, an examination of the normalized outcome values (ratios) revealed that they were bounded at zero and highly skewed. Thus, we relied on bootstrap-based standard errors for inference. The bootstrap was based on 118 clusters (118 subjects) and 500 replicates. The bootstrap provided variance estimates for each of the regressions. Hypotheses were tested at a type one error of 0.05, using z-scores generated using the bootstrap standard errors. Hypotheses related to sex differences (main effects at day 8 and trends by sex from day 8 to day 21) were tested using the z-score associated with the regression parameters. A small number of variables found to be associated with sex (at p < 0.1) were included as covariates in the model. Analyses were conducted using Stata version 18.

## 3. Results

### 3.1. Sample characteristics

A total of 141 adults attended the baseline visit and enrolled in the study. Among these, 23 participants were excluded from the analysis; 2 were withdrawn for not following procedures, and 21 withdrew from the study due to lack of interest and time or were lost at different time points during the switch phase, resulting in incomplete data. The analytic sample comprised 118 participants. The sample had slightly more males (60.0 %) than females and had an average age of 51.3 years (SD=10.6) and 52.4 years (SD=8.5), respectively. Most reported their race as either White (38.1 %; males = 42 %, females = 32 %) or Black (56.8 %; males = 51.0 %, females = 66.0 %). Both males and females tended to smoke menthol cigarettes, 67.6 % and 76.6 %, respectively. Males and females were moderately nicotine dependent (M = 4.96, SD =1.8; M = 5.19, SD =2.0, respectively) and smoked 14.30 CPD (SD = 6.53) and 12.83 CPD (SD = 6.71) during the 5-day ad-lib baseline smoking period, respectively. There were no significant sex differences for any of these variables. See Table 1.

### 3.2. Do the effects of IQOS use on craving, withdrawal, subjective reward, and relative reinforcement differ by sex?

As noted in Table 1, withdrawal symptoms were low for males and females after 10 h of overnight abstinence and were not significantly different (p = 0.09). Cigarette cravings were moderate and greater for females than males for the positive subscale (p = 0.06) and the negative subscale (p = 0.011) following the 10-hour overnight smoking abstinence. Neither males nor females reported significant changes in craving or withdrawal after IQOS exposure (p > 0.05). In addition, the use of IQOS did not differentially reduce cigarette cravings or withdrawal symptoms (p values > 0.05). IQOS use was moderately rewarding for males and females (p = 0.32). During the relative reinforcement task, males and females worked on an average of 7.5 trials for cigarette puffs, indicating that, on average, both found smoking more reinforcing than IQOS (p = 0.99). Relative to IQOS, 78 % of males and 83 % of females worked predominately for cigarette puffs (≥ 5 trials; (Epstein et al., 2004)), while 22 % of males and 17 % of females were far less willing to work for cigarette puffs (< 5 trials), p = 0.53.

### 3.3. Does IQOS use impact cigarette smoking differently by sex?

**Fig. 1.**
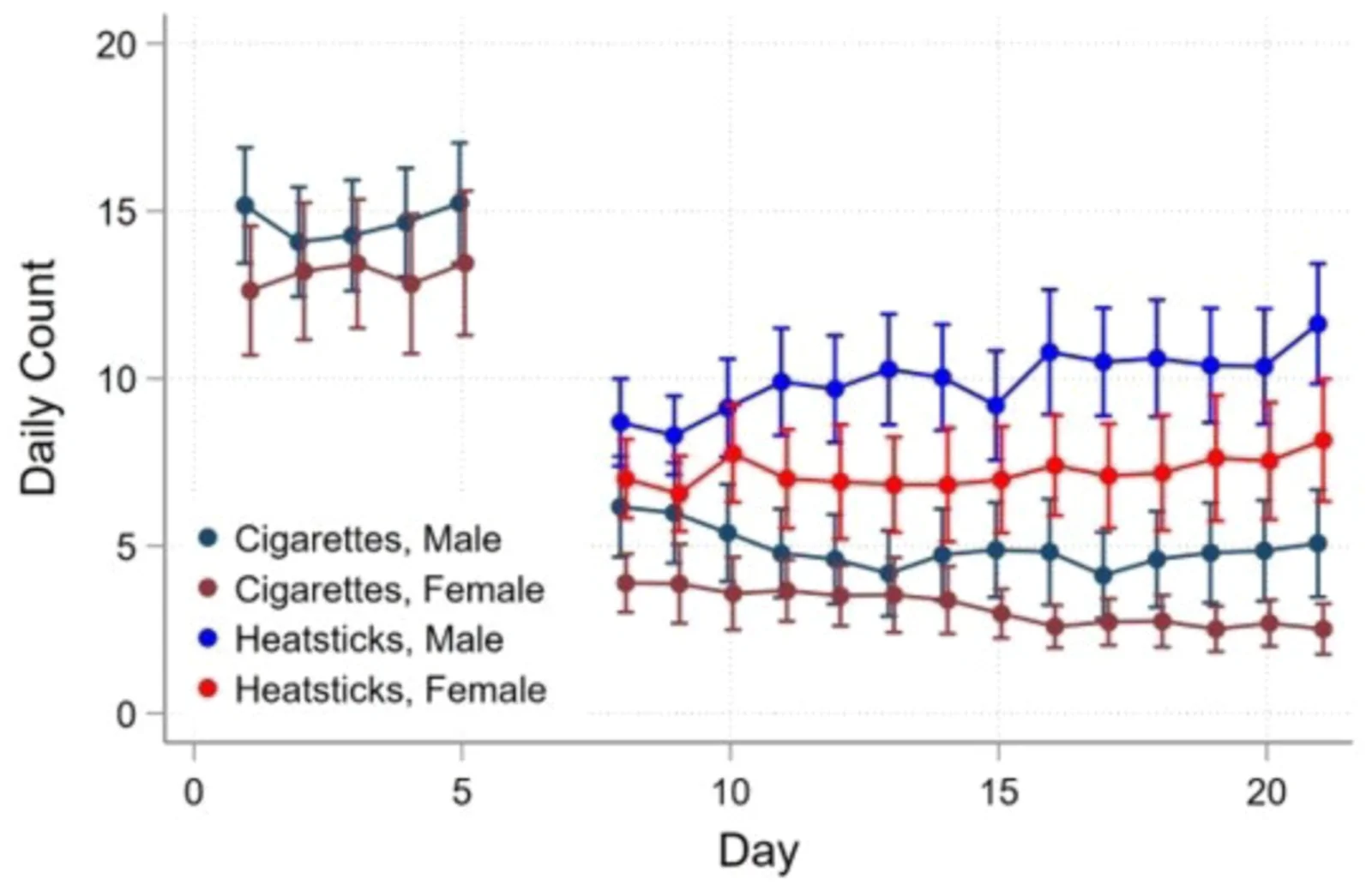
Number of cigarettes smoked and IQOS HeatStick used by sex.

Females decreased their CPD from a baseline average of 12.83 CPD to an average of 3.72 CPD on day 8 (t(46) = −9.27, p > 0.0001), decreasing to an average of 2.49 CPD on day 21 (t(46) = −2.31, p > 0.026). Males decreased their CPD from a baseline average of 14.30 CPD to an average of 6.00 CPD on day 8 (t(70) = −12.32, p > 0.0001), decreasing to an average of 5.03 CPD on day 21 (t(70) = −1.55, NS). During the switch period, daily IQOS use increased for females from an average of 6.85 HeatSticks (95 %CI = [5.09, 8.62]) per day at day 8–8.13 HeatSticks (95 %CI = [6.36; 9.89]) per day at day 21 (t(46) = 1.24, NS). During the switch period, daily IQOS use increased for males from an average of 8.49 HeatSticks (95 %CI = [7.06, 9.93]) per day at day 8–11.24 HeatSticks (95 %CI = [9.80; 12.66]) per day at day 21 t(70) = 3.75, p > 0.001). See Fig. 1.

Thirteen percent of females and 23 % of males fully switched to IQOS, defined as a reduction to 0 CPD throughout the last 7 days of the switch period, biochemically validated by a CO ≤ 8; χ^2^(1) = 1.88, p = .20. Females had a baseline CO of 16 ppm and males 15 ppm. The average CO among females and males who fully switched was 2.67 ppm and 3.62 ppm, respectively, and the average CO among those who did not was 9.60 ppm and 10.74 ppm.

**Fig. 2.**
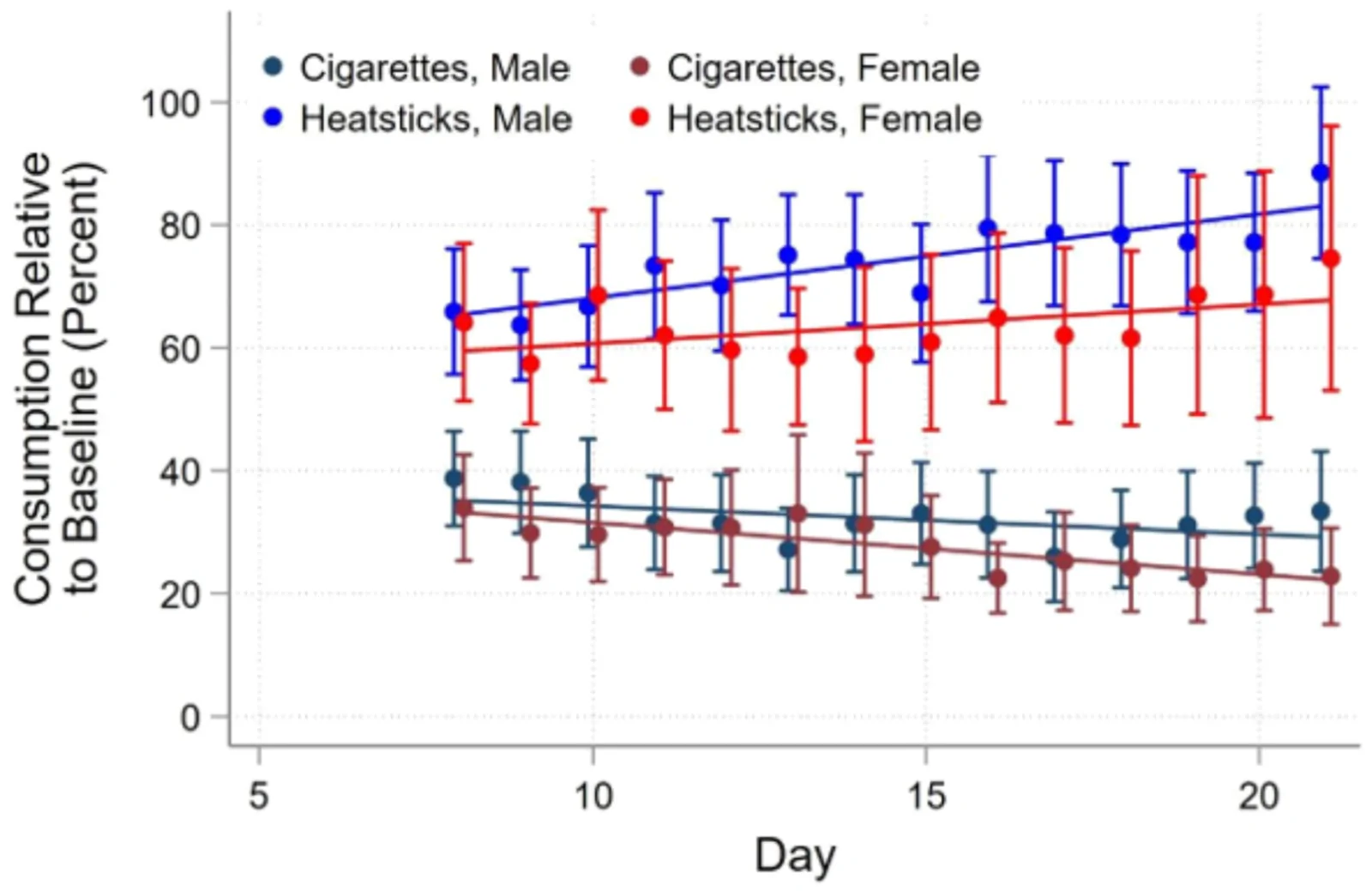
Changes in cigarette smoking and IQOS HeatStick use by sex.

In the covariate-adjusted model (Table 2, Fig. 2), smoking was ~35.8 % of baseline CPD (Predicted Mean=35.8 [95 %CI 28.6–42.9] p < 0.001 tested against 100 %) for males and 32.5 % for females at day 8 (Predicted Mean=32.5 % [95 %CI 25.5–39.6] p < 0.001 tested against 100 %). Cigarette smoking continued to decline linearly from day 8–21 for both males (β=−0.46 [95 %CI −0.97–0.05] p = 0.07) and females (β=−0.83 [95 %CI −1.34 to −0.32] p = 0.002). Baseline positive and negative craving and CPD did not predict smoking during the switch period (p values >0.05). The interaction between sex and baseline craving was not significant (p = 0.16 for negative craving and p = 0.19 for positive craving). Baseline withdrawal symptoms (β=1.0 [95 %CI 0.14–1.86] p = 0.023) and pre-post IQOS exposure changes in the negative craving subscale predicted increased smoking reduction (β=1.71 [95 %CI 0.40–3.03] p = 0.011). The interaction between sex and changes in negative craving was not significant (p = 0.94).

### 3.4. Does IQOS substitution for cigarettes differ by sex?

The IQOS substitution rate served as the outcome variable for this analysis. On day 8, IQOS use (Table 3, Adjusted model, Fig. 2) was approximately 65.6 % of baseline CPD (Predicted Mean = 65.6 [CI95 % 56.6–74.3] p < 0.001) for males. Replacement was slightly lower for females (Predicted Mean =59.2 % [CI95 % 50.5–67.9] p = 0.43) than males. IQOS use continued to climb by 1.36 % per day for males (β = 1.36 [CI95 % 0.59–2.14] p = 0.001) and 0.64 % per day for females (β = 0.64 [CI95 % −0.67–1.94] p = 0.34); the slopes did not differ significantly from each other (χ^2^(1) = .83, p = .36). Neither baseline withdrawal symptoms nor any craving measure predicted substitution during the switch period (P values >0.05). However, baseline CPD predicted decreased substitution (β=−2.19 [95 %CI −3.33 to −1.04] p < 0.001).

## 4. Discussion

This study is the first to investigate sex differences in IQOS-associated effects and the impact of IQOS use on cigarette smoking. IQOS use was associated with a 78 % reduction in cigarette smoking for females and a 70 % reduction in cigarette smoking for males. Males and females similarly substituted IQOS HeatSticks for their cigarettes − 83 % and 67 %, respectively. Females and males did not differ in IQOS reward, reinforcement, or cigarette abstinence symptom relief. These findings suggest that harm-reduction products, such as IQOS, that mimic the behavioral and sensory features of cigarette smoking and deliver nicotine have the potential to similarly aid females and males who have been unable to quit smoking with traditional smoking cessation medication.

Females and males who smoke cigarettes daily significantly reduced their daily smoking rate with IQOS use, and a meaningful subset completely switched to IQOS. Research has documented that different aspects of smoking reduction, such as the magnitude and duration of the reduction, bolster the likelihood of subsequent cessation (Klemperer and Hughes, 2016). Replacing one’s cigarettes with IQOS may foster motivation for further smoking behavior change. Indeed, our research has shown that IQOS use is associated with increased motivation to quit smoking (Audrain-McGovern et al., 2025). The sample comprised adults not planning to quit smoking in the immediate future. Yet, males and females fully switched from cigarette smoking to IQOS use (23 % and 13 %, respectively) within the 14-day switch period, although males slightly more than females. The CO among females who reduced their smoking rate decreased by 40 %, and among females who ceased smoking, it decreased by 83 %. The CO among males who reduced their smoking rate decreased by 28 %, and among males who fully switched to IQOS decreased by 76 %. These changes in CO provide evidence for less exposure to combustion-based toxicants, particularly among those who fully switched. Further research is necessary to measure the reduction in exposure to specific toxicants when switching is complete or incomplete and to determine if it differs between males and females.

As the first independent study to assess sex differences in the effects of IQOS on smoking behavior, we can only compare the present findings to one tobacco industry-sponsored study (n = 965). Roulet and colleagues found no difference between men and women in IQOS adoption (17 % versus 12 %, respectively) across six weeks of ad-libitum use without instructions to switch (Roulet et al., 2021). Adoption was defined as using HeatSticks for at least 70 % of all tobacco products used. While the outcome was not CO-verified, the lack of sex differences in a larger sample with a longer use period is consistent with our findings.

Males and females similarly substituted IQOS HeatSticks for their cigarettes − 83 % and 67 %, respectively. Females smoked almost 13 cigarettes per day at baseline, replaced approximately eight with HeatSticks, and continued to smoke ~2.5 cigarettes per day, indicating that two cigarettes were not replaced. This pattern of use may suggest that the nonpharmacological features of using IQOS (e.g., mimic behavior of smoking, sensations of inhalation and exhalation) outweighed the nicotine replacement that would accompany greater HeatStick or cigarette use. In contrast, males smoked almost 14 cigarettes per day at baseline, replaced approximately 11 with HeatSticks, and continued to smoke five cigarettes per day, indicating a surplus of two cigarettes. The pattern of IQOS and cigarette use among males suggested that they were more sensitive to maintaining nicotine levels, as IQOS nicotine delivery is less than that of combustible cigarettes (Maloney et al., 2020; Vukas et al., 2023). Research has shown that females were more likely to quit smoking than males when assigned to a very low-nicotine cigarette, yet males were more likely than females to quit when assigned to the nicotine patch (Vogel et al., 2014).

Finally, males and females did not differ in IQOS-associated effects. Both found IQOS rewarding, although less reinforcing relative to their cigarettes, which is consistent with research on IQOS-associated effects that did not consider sex as a source of individual differences (Adriaens et al., 2018; Funk et al., 2023; Maloney et al., 2020; Yingst et al., 2024). Withdrawal symptoms and craving showed slight and comparable decreases for both males and females after one HeatStick exposure. Studies of the acute effects of IQOS after overnight abstinence have shown that IQOS use reduces cigarette cravings while the effects on withdrawal symptom relief are mixed (Adriaens et al., 2018; Maloney et al., 2020; Stone et al., 2022; Yingst et al., 2024).

As the first study of sex differences in the substitutability of IQOS for combustible cigarette smoking, the study has strengths and limitations. Strengths include measuring the positive and negative reinforcing effects of IQOS with validated laboratory paradigms and assessing their impact on subsequent cigarette smoking and IQOS use in the participants’ typical environment. Cigarette smoking and IQOS use were verified via spent product collection and CO assessment. Finally, we recruited and retained a diverse sample of adults who smoke.

One potential limitation is that the two-week switch period may not have provided sufficient time for a sex difference in switching or cigarette reduction to emerge. While research has shown that changes in cigarette smoking during the initial two weeks are predictive of subsequent smoking behavior (Donny et al., 2015; Mercincavage et al., 2018; Nollen et al., 2023; Sargent et al., 2024), this can only be confirmed with a longer-term follow-up. Similarly, without a follow-up, we cannot document whether exclusive use or the level or presence of dual use with combustible cigarettes changes for males and females. At the end of the 14-day switch period, females who had not fully switched were smoking about 3 cigarettes per day (22 % of their baseline smoking rate), and males who had not fully switched were smoking about five cigarettes per day (30 % of their baseline smoking rate). Unfortunately, we are unable to determine whether these significant reductions in cigarette smoking subsequently resulted in cigarette cessation, continued dual use, or a return to exclusive cigarette smoking.

In addition, without a non-nicotine HeatStick comparison, we can state that IQOS use results in comparable outcomes for females and males, but we can only speculate that the motivations underlying the outcomes are different, nonpharmacological and pharmacological, respectively. Similarly, without a comparison to another harm-reduction product (e.g., e-cigarettes), it is unclear whether the observed effects are specific to IQOS or reflect harm-reduction products in general. Future research should consider the effects of IQOS alongside another nicotine delivery product to strengthen the inferences regarding the specificity of IQOS effects.

Finally, the effect we observed in this secondary data analysis was small (0.18), indicating a sample size of ~950 participants would be required to detect a significant difference between males and females in cigarette smoking. The question is whether an 8 % difference in cigarettes per day for females versus males is a clinically meaningful difference. It is likely not. While the difference between males and females in cigarettes per day was modest, we cannot assume that the lack of statistical difference is evidence of equivalence.

In conclusion, this study did not find significant sex differences in IQOS-associated effects or the initial substitutability of IQOS for combustible cigarette smoking. For harm-reduction approaches to advance empirically, sex differences in the response to alternative nicotine delivery products warrant examination. Such information may help to optimize the transition away from combustible cigarettes among females and males who have been unable with current smoking cessation medications or are disinterested in their use.

## Figures and Tables

**Table 1:** Effects of IQOS by sex (N = 118).

Variable	Male N (%) or Mean (SD)	Female N (%) or Mean (SD)	P-value
N (%)	71 (60 %)	47 (40 %)	
Race			0.19
Black/ African American	36 (51 %)	31 (66 %)	
White	30 (42 %)	15 (32 %)	
Other	5 (7 %)	1 (2 %)	
Age	51.30 (10.62)	52.43 (8.54)	0.54
Baseline Cigarettes Per Day	14.33 (6.53)	12.83 (6.71)	0.23
Nicotine Dependence (FTND)	4.96 (1.77)	5.19 (2.01)	0.51
Subjective Reward (CES)	8.27 (2.80)	7.70 (3.31)	0.32
Breakpoint	7.52 (3.53)	7.53 (3.17)	0.99
Breakpoint (dichotomized)			0.53
< 5	15 (22 %)	8 (17 %)	
≥ 5	54 (78 %)	39 (83 %)	
Baseline Withdrawal Symptoms	8.05 (5.53)	9.93 (5.84)	0.09
Withdrawal Relief (MNWS)	−0.70 (3.23)	−0.91 (4.11)	0.76
Baseline Craving (QSU Negative)	10.06 (5.79)	13.18 (6.86)	0.01
Baseline Craving (QSU Positive)	26.36 (7.29)	29.20 (8.06)	0.06
Craving Relief (QSU Negative)	−2.54 (5.09)	−4.27 (5.79)	0.10
Craving Relief (QSU Positive)	−8.82 (8.46)	−9.22 (8.68)	0.81

**Table 2:** Sex differences in IQOS effects on cigarette smoking during 14-day switch period.

Cigarette Smoking - % of average baseline CPD					Bootstrapped Standard Errors		
Variable	Coefficient	Std. error	p-value	95 % CI	Std. error	p-value	95 % CI
Female	−3.21	5.35	0.549	−13.71, 7.28	5.15	0.533	−13.29, 6.87
Male × Time	−0.46	0.15	0.002	−0.75, −0.17	0.26	0.073	−0.97, 0.04
Female × Time	−0.83	0.18	0.000	−1.18, −0.47	0.26	0.002	−1.34, −0.31
Baseline Withdrawal (MNWS)	0.99	0.43	0.020	0.16, 1.84	0.44	0.023	0.14, 1.86
Baseline Craving (QSU Negative)	0.55	0.62	0.369	−0.65, 1.76	0.66	0.399	−0.73, 1.84
Baseline Craving (QSU Positive)	0.13	0.42	0.755	−0.69, 0.95	0.42	0.757	−0.69, 0.95
Craving Relief (QSU Negative)	1.71	0.61	0.005	0.52, 2.90	0.67	0.011	0.39, 3.02
Baseline Cigarettes per day	−0.23	0.38	0.544	−0.98, 0.52	0.44	0.595	−1.09, 0.62
Constant	25.90	10.02	0.010	6.26, 45.55	9.82	0.008	6.66, 45.15
Test of equal slopes for males and females		χ^2^(1) = 2.40, p = .12			χ^2^(1) = 1.03, p = .31		

Note. MNWS, Minnesota Nicotine Withdrawal Scale (Likert response options: 0 =None to 4 =Severe; range=0–32; α=0.87). The difference score reflects post-exposure minus pre-exposure. QSU, Questionnaire of Smoking Urges (Likert response options: 1 =Strongly Disagree to 7 =Strongly Agree; QSU Negative range= 4–28, α=0.81; QSU Positive range= 5–35, α=0.91). The difference score reflects post-exposure minus pre-exposure.

**Table 3:** Sex differences in IQOS substitution rate across the 14-day switch period.

IQOS Substitution Rate - HeatStick use normalized as % of baseline CPD					Bootstrapped Standard Errors		
Variable	Coefficient	Std. error	p-value	95 % CI	Std. error	p-value	95 % CI
Female	−6.28	8.01	0.433	−21.98, 9.41	6.78	0.354	−19.57, 7.00
Male × Time	1.36	0.21	0.000	0.95, 1.78	0.40	0.001	0.59, 2.14
Female × Time	0.64	0.26	0.014	0.13, 1.14	0.66	0.339	−0.67, 1.94
Baseline Withdrawal (MNWS)	−0.04	0.64	0.957	−1.30, 1.23	0.62	0.955	−1.25, 1.18
Baseline Craving (QSU Negative)	−1.10	0.93	0.237	−2.91, 0.72	0.93	0.236	−2.91, 0.72
Baseline Craving (QSU Positive)	0.15	0.63	0.813	−1.08, 1.37	0.53	0.780	−0.89, 1.19
Craving Relief (QSU Negative)	−0.09	0.92	0.925	−1.88, 1.71	0.93	0.926	−1.90, 1.73
Baseline Cigarettes per day	−2.19	0.57	0.000	−3.31, −1.06	0.58	0.000	−3.33, −1.04
Constant	104.50	15.05	0.000	75.00, 133.99	13.71	0.000	77.63, 131.37
Test of equal slopes for males and females		χ^2^(1) = 4.80, p = .03			χ^2^(1) = .83, p = .36		

*Note*. MNWS, Minnesota Nicotine Withdrawal Scale (Likert response options: 0 =None to 4 =Severe; range=0–32; α=0.87). The difference score reflects post-exposure minus pre-exposure. QSU, Questionnaire of Smoking Urges (Likert response options: 1 =Strongly Disagree to 7 =Strongly Agree; QSU Negative range= 4–28, α=0.81; QSU Positive range=5–35, α=0.91). The difference score reflects post-exposure minus pre-exposure.
